# Modeling network phenomena in the Inferior Olive: II. Modulation of sub-threshold oscillations

**DOI:** 10.1186/1471-2202-12-S1-P380

**Published:** 2011-07-18

**Authors:** Benjamin Torben-Nielsen, Yaara Lefler, Idan Segev, Yosef Yarom

**Affiliations:** 1 Edmond and Lily Safra Center for Brain Sciences, Hebrew University, Jerusalem, Israel; 2 Interdisciplinary Center for Neural computation, Hebrew University, Jerusalem, Israel; 3 Department of Neurobiology, Hebrew University, Jerusalem, Israel

## 

Sub-threshold oscillations (STOs) provide a timing signal for the Inferior Olive (IO) neurons as IO cells are known to fire only phase-locked to the peak of the STO. Moreover, based on in-vitro and in-vivo experiments, it has been proposed that the STOs are actively modulated to represent spatiotemporal patterns [1,2]. Modulations are manifested in changes in frequency and amplitude, and a stable phase-shift between different clusters to accommodate for timing signals with a higher temporal resolution than the period of the oscillation [3].

In this work we investigated how STOs can be dynamically modulated. To this end we simulated networks of canonical IO neurons including a low-threshold Ca^2+^ current and a leak current. In isolation, these cells can generate spontaneous oscillations at different frequencies (4-17 Hz) for particular densities of the associated g_Ca_ and g_L_ conductances [4]. When coupled through gap-junctions, a population of such neurons with heterogeneous densities of g_Ca_ and g_L_ will -in general- exhibit spontaneous STOs as long as the “average cell” (i.e., a weighted average of the conductances of all connected cells) would be a spontaneously oscillating cell. Thus, in theory, the frequency of the oscillations can be directly modulated by changing the “average cell”, i.e., by manipulating the contributions of each cell to the “average cell”. In practice, the contribution of a cell is related to the coupling strengths to other cells. Hence, increasing and decreasing the coupling strength between cells changes the “average cell” and causes a change in frequency of the STOs. We simulated networks with a realistic number of cells (for a given amount of tissue) and changed the coupling strengths during the simulation. In some cases we simulated a complete decoupling between different parts of the network. We found that for a range of realistic coupling strengths we could reproduce two experimental observations: Sudden jumps in STO frequency between 6-12 Hz, and, stable phase-shifts of up to 30 ms between (self-organizing) clusters within the simulated networks.

We argue that the GABAergic inputs from the deep cerebellar nucleus (DCN) are well suited to adjust the coupling strength as they are known to shunt gap-junctional currents when activated. Also, we speculate that the stable phase-shift between clusters causes the observed “waves” of activation in IO neurons [1]. An alternative mechanism that can modulate the STO frequency is based on activity-dependent changes in the Ca^2+^ driving force which we found to affect the frequency of the STO in simulations. We conclude that changing the coupling strengths of the network by GABAergic inputs from the DCN is a plausible mechanism to actively modulate spatiotemporal patterns.
